# HPV-Associated Field Cancerization: Molecular Alterations in Histologically Normal Tissues and Implications for Early Detection and Cancer Prevention

**DOI:** 10.3390/cimb48090932

**Published:** 2026-09-11

**Authors:** Atakan Demir, Güliz Avşar, Begüm Kurt, Muharrem Okan Cakır, Hossein Ashrafi

**Affiliations:** 1 Department of Medical Oncology, Memorial Goztepe Cancer Center, Istanbul 34730, Turkey; atakan.demir@memorial.com.tr; 2 Department of General Surgery, Faculty of Medicine, Akdeniz University, Antalya 07070, Turkey; guliz989@gmail.com; 3 Department of Biochemistry, School of Medicine, Marmara University, Istanbul 34854, Turkey; begumkurt2002@gmail.com; 4 School of Life Sciences, Pharmacy and Chemistry, Kingston University London, London KT1 2EE, UK

**Keywords:** human papillomavirus, field cancerization, cervical cancer, oropharyngeal cancer, anal cancer, bladder cancer, DNA methylation, liquid biopsy

## Abstract

Human papillomavirus (HPV)-associated cancers have traditionally been viewed as focal diseases arising from localized malignant transformation. However, growing molecular evidence suggests that HPV-associated molecular alterations may extend beyond visible lesions into histologically normal epithelium, raising the possibility of a virus-associated field effect. In this review, HPV-induced field cancerization is distinguished from HPV infection alone and is used to describe spatially extended molecular abnormalities demonstrated in histologically normal HPV-positive or tumor-adjacent tissue, whereas findings from premalignant lesions, established tumors, and experimental models are considered supportive evidence of progression or mechanism. This emerging concept challenges conventional models of HPV-driven carcinogenesis and carries important implications for early detection and cancer prevention. Evidence relevant to HPV-associated field effects includes spatially distributed viral, epigenetic, transcriptomic, and immune alterations in selected histologically normal tissues, while findings related to viral integration, metabolic reprogramming, premalignant lesions, tumors, and experimental models provide complementary progression or mechanistic evidence. Such molecular abnormalities have been reported most consistently in cervical disease, with supportive but more limited evidence in the oropharynx and anal canal, whereas the relevance of HPV-associated field effects in the urinary bladder remains controversial. In this review, we aim to synthesize current molecular evidence supporting HPV-induced field cancerization, discuss potential underlying mechanisms, and evaluate emerging methodological approaches for detecting field effects in normal tissues. We further highlight the translational relevance of this phenomenon for molecular-based screening, risk stratification, and preventive strategies aimed at intercepting HPV-associated cancers at their earliest stages.

## 1. Introduction

Human papillomavirus (HPV) is responsible for a substantial share of the global cancer burden. According to GLOBOCAN 2022, the most recent global cancer estimates available, cervical cancer accounted for approximately 662,000 new cases and 349,000 deaths worldwide and remained the fourth most commonly diagnosed cancer among women [1]. For decades, HPV-associated carcinogenesis has been conceptualized as a focal process in which malignant transformation arises at a single, discrete anatomical point and expands locally from that point, an assumption that has shaped screening algorithms, surgical margins, and post-treatment surveillance protocols across cervical, oropharyngeal, and anal oncology. This focal model is increasingly difficult to reconcile with molecular observations demonstrating that epithelium located well beyond the boundary of any visible or histologically defined lesion frequently harbors alterations that closely resemble those found within the tumor itself.

The idea that carcinogenesis extends beyond a clinically apparent lesion was first articulated more than seventy years ago by Slaughter and colleagues, who examined resected specimens from 783 patients with head and neck squamous cell carcinoma and observed epithelial atypia at the margins of virtually every tumor studied, a phenomenon the authors termed field cancerization [2]. Their original formulation proposed that oral cancer develops from multifocal areas of preneoplastic change, that tissue surrounding the tumor is histologically abnormal, that lesions are frequently multiple and may coalesce, and that persistence of abnormal tissue after surgical resection can account for local recurrence and second primary tumors. Although this concept was originally developed in the context of tobacco- and alcohol-related carcinogenesis, subsequent molecular work has raised the possibility that analogous field processes may operate in selected HPV-driven malignancies, although the strength of evidence varies considerably according to the anatomical site [3]. This review synthesizes current molecular evidence supporting the existence of HPV-induced field cancerization, examines the mechanisms proposed to underlie this phenomenon, and evaluates the methodological approaches now available to detect field effects in tissue that appears entirely normal by conventional histopathology. Particular attention is given to the translational relevance of this evidence for molecular-based screening, risk stratification, and strategies aimed at intercepting HPV-associated cancers before overt neoplastic transformation occurs.

## 2. Materials and Methods

This article was prepared as a narrative review. The methodology described here was designed to improve the transparency and reproducibility of the literature search rather than to meet the requirements of a systematic review or meta-analysis. Relevant publications were identified through searches of PubMed/MEDLINE, Scopus, and Web of Science Core Collection, with the final search conducted in August 2026.

The search strategy combined terms related to human papillomavirus (HPV) with terms describing field cancerization, including “field cancerization,” “field effect,” “histologically normal tissue,” “normal appearing epithelium,” “adjacent tissue,” “peritumoral mucosa,” and “molecular field.” Additional searches incorporated terms related to the major molecular and diagnostic themes addressed in this review, including DNA methylation, epigenetic and transcriptomic alterations, single-cell and spatial transcriptomics, metabolic and immune microenvironment changes, viral integration, liquid biopsy, circulating HPV DNA, cell-free HPV DNA, early detection, and screening. These terms were further combined with anatomical site descriptors including cervix, cervical, oropharynx, head and neck, anal canal, and bladder.

Only English-language publications were considered. Eligible studies included peer-reviewed original research, translational and experimental studies, cohort and case control studies, and systematic reviews and meta-analyses. Mechanistic and narrative reviews were used primarily to support background information and conceptual interpretation rather than as primary evidence. Case reports, conference abstracts, letters, editorials, non-peer-reviewed material, and studies not directly relevant to HPV-associated field effects were excluded. Reference lists of key publications were also screened to identify additional relevant studies. The review was prepared with consideration of the Scale for the Assessment of Narrative Review Articles (SANRA) framework to improve methodological transparency. Because the article is a narrative rather than a systematic review, it was not structured according to the Preferred Reporting Items for Systematic Reviews and Meta Analyses (PRISMA) framework.

To distinguish field cancerization from HPV infection alone or from conventional neoplastic progression, the included evidence was also categorized according to biological context. Studies were grouped as: (i) histologically normal HPV-positive human tissue, (ii) histologically normal tumor-adjacent or peritumoral human tissue, (iii) premalignant lesions, (iv) established tumors, and (v) experimental cell-line or organoid models. Findings from histologically normal HPV-positive or tumor-adjacent human tissues were considered capable of providing direct evidence of a field effect when spatial extension, multifocality, or molecular continuity with an associated lesion was demonstrated. In contrast, findings derived exclusively from premalignant lesions, established tumors, cell lines, or organoid models were interpreted as supportive evidence of disease progression or biological mechanism rather than as direct proof of field cancerization.

## 3. HPV Biology and the Classical Model of Focal Carcinogenesis

### 3.1. HPV Attachment and Entry Factors in Normal and Neoplastic Epithelium

HPV infection is initiated through access to basal epithelial keratinocytes, typically following disruption of the epithelial barrier. Viral attachment involves interactions with heparan sulfate proteoglycans on epithelial cells and subsequent engagement of cell-associated factors that facilitate internalization [3]. Following entry, the viral genome can be maintained within basal epithelial cells, allowing for persistent infection and subsequent HPV-associated cellular reprogramming [3].

Importantly, the molecular studies included in this review do not establish a specific HPV surface receptor signature that consistently distinguishes normal from premalignant or malignant epithelial cells. Single-cell and spatial transcriptomic studies demonstrate substantial changes in epithelial cell states across HPV-infected normal tissue, precancer, and cancer, while experimental HPV16-infected epithelial models similarly identify reprogrammed keratinocyte populations. These findings indicate progressive remodeling of epithelial phenotype but should not be interpreted as evidence that differential expression of a particular HPV entry receptor defines the transition from normal to cancerous tissue.

### 3.2. Oncoprotein-Driven Transformation

High-risk HPV genotypes encode the oncoproteins E5, E6, and E7, which act cooperatively to prolong cell cycle progression, delay terminal differentiation, and suppress apoptosis in infected keratinocytes, thereby generating a cellular environment permissive of viral replication [4]. The E7 oncoprotein binds and promotes functional inactivation of the retinoblastoma tumor suppressor protein, releasing E2F transcription factors and driving inappropriate entry into S phase, while the E6 oncoprotein recruits the E3 ubiquitin ligase E6AP to mediate proteasomal degradation of p53, thereby disabling the principal checkpoint that would otherwise trigger cell cycle arrest or apoptosis in response to the DNA damage generated by unscheduled proliferation [5]. Together, functional inactivation of these two tumor suppressor pathways establishes a cellular state in which host genomic instability can accumulate over years of persistent infection, providing the substrate upon which secondary genetic and epigenetic alterations are subsequently selected [4,5].

### 3.3. Viral Integration and Genomic Consequences

A further hallmark of HPV-driven transformation is integration of the circular viral episome into the host genome, an event that occurs in a substantial proportion of high-grade lesions and invasive cancers and that typically disrupts the viral E2 open-reading frame, releasing E6 and E7 from E2-mediated transcriptional repression and permitting their sustained overexpression [6]. Integration is now understood to proceed largely through microhomology-mediated end joining and related double-strand break repair pathways, frequently targeting recurrent fragile regions of the host genome such as chromosomal bands 3q28 and 8q24, and is followed by a cascade of secondary events, including local genomic rearrangement, formation of extrachromosomal DNA, and epigenetic remodeling of chromatin surrounding the integration site [6,7]. Because viral integration can occur relatively early during HPV-associated neoplastic progression, integration-related genomic alterations provide a plausible mechanism through which altered clones could expand before invasive cancer develops [6]. However, the demonstration of HPV integration within a premalignant lesion or tumor does not by itself establish field cancerization; direct field evidence requires demonstration of such alterations within spatially extended, histologically normal epithelium.

Importantly, viral integration is not a prerequisite for HPV-associated molecular remodeling. High-risk HPV genomes can persist episomally in basal keratinocytes during persistent infection, allowing for viral gene expression and host cell reprogramming before or in the absence of integration [3,4,5]. Integration should therefore be regarded as one mechanism that may reinforce clonal selection and genomic instability during neoplastic progression rather than as a necessary initiating event for field formation [6]. This distinction also helps differentiate HPV-associated field processes from classical carcinogen-driven field cancerization. Broad exposure to carcinogens such as tobacco or alcohol may generate multiple independently altered epithelial clones across an exposed surface, whereas an HPV-associated field may arise through persistent infection at multiple epithelial foci and/or expansion of an infected or transformed clone [2,3]. These mechanisms are not mutually exclusive, and their relative contribution may vary according to anatomical site and stage of disease.

## 4. From Slaughter to HPV: Conceptual and Operational Definition of Field Cancerization

The classical field cancerization framework proposed by Slaughter and colleagues rested entirely on histopathological observation, namely the recognition of epithelial atypia adjacent to resected tumors [2]. The molecular era has reframed this concept in mechanistic terms, distinguishing a classical model, in which chronic carcinogen exposure independently damages a broad epithelial surface, from a clonal model, in which a single altered cell or small population of cells acquires a growth advantage and progressively displaces the surrounding normal epithelium, generating a field of clonally related, molecularly altered but histologically unremarkable tissue. In tobacco- and alcohol-related head and neck cancer, this clonal field has been mapped using shared molecular markers, including hypermethylation of CDKN2A and MGMT together with dysregulation of MDM2, E2F2, ETS-1, and multiple microRNAs, which are detectable in peritumoral mucosa at frequencies approaching those found within the tumor itself, indicating that the biologically altered field extends well beyond the histologically defined tumor margin [8]. Field cancerization is therefore not specific to HPV-associated malignancies or to a single carcinogenic exposure. Rather, it represents a broader biological concept in which molecularly altered epithelial cells extend beyond the boundaries of a focal lesion. Such fields may arise through chronic carcinogen exposure, clonal expansion, persistent oncogenic infection, or a combination of these processes. The defining feature is the presence of molecular alterations beyond the clinically or histologically apparent lesion, rather than the identity of a particular cancer type or carcinogenic trigger [2,3].

In this review, HPV-associated field cancerization refers to molecular alterations that extend beyond a defined lesion into histologically normal HPV-positive, tumor-adjacent, or peritumoral epithelium. Evidence was considered more consistent with a field effect when these abnormalities showed spatial extension, multifocal distribution, or molecular similarity to an associated lesion. HPV positivity alone was not considered sufficient to establish field cancerization. Findings limited to premalignant lesions or established tumors were therefore interpreted primarily as evidence of HPV-associated disease progression, while observations from cell-line and organoid models were regarded as mechanistic support rather than direct demonstration of a molecular field in human tissue.

Because HPV directly reprograms the keratinocytes it infects through the oncoproteins described above, the virus provides an alternative and biologically distinct route to field formation, one that does not require an external chemical carcinogen but instead depends on the capacity of the virus to establish persistent infection across a contiguous or multifocal epithelial surface and to drive clonal expansion of infected or transformed cells within that surface [3]. Experimental work in cutaneous and mucosal models has shown that HPV early genes can reprogram adult tissue stem cells, expanding their self-renewal capacity and displacing them from their normal niche, a mechanism proposed to underlie the multifocal presentation characteristic of HPV-associated intraepithelial neoplasia across several organ systems [3]. This mechanistic convergence, in which a virus achieves an outcome structurally analogous to that produced by chemical carcinogens in the classical field cancerization model, forms the conceptual bridge between the original observations published in 1953 and the molecular findings reviewed in the sections that follow.

## 5. Molecular Alterations Underlying HPV-Induced Field Cancerization

### 5.1. Persistent Viral Infection and Multifocality in Clinically Normal Tissue

Direct evidence for viral persistence beyond the margins of overt disease has been generated through three-dimensional mapping studies that combine radiological imaging with molecular sampling of the mucosa surrounding head and neck tumors. In one such study, biopsy mapping of the mucosal field surrounding oropharyngeal squamous cell carcinoma demonstrated that peritumoral tissue exhibited patchy but reproducible alterations in oncogenic microRNA expression, including miR-21-5p and miR-155, that differed according to the HPV status of the underlying tumor, indicating that the molecular field surrounding an HPV positive tumor is qualitatively distinct from that surrounding an HPV-negative, carcinogen-driven tumor and extends into tissue that appears histologically unremarkable [9]. These findings support the use of combined radiological and molecular mapping to define the true extent of the biologically altered field, information that has direct relevance for surgical margin planning and organ-preserving therapy [9].

### 5.2. Epigenetic Dysregulation and DNA Methylation Fields

Aberrant DNA methylation is among the earliest and most extensively characterized molecular alterations associated with HPV-driven carcinogenesis and provides a particularly informative marker of field effects, because methylation changes are stable and quantifiable and can be detected across different stages of HPV-associated neoplastic progression, including in samples without overt morphological atypia. Importantly, prediagnostic evidence is also available: in a prospective cohort, increased HPV16 methylation in cervical specimens collected before cervical intraepithelial neoplasia grade 3 or worse (CIN3) diagnosis was associated with subsequent cervical precancer [10]. Genome-wide methylation profiling of anal and cervical tissue spanning the full spectrum from normal epithelium through high-grade intraepithelial neoplasia to invasive carcinoma has identified partial least-squares signatures capable of distinguishing normal from malignant tissue at both sites, with seventeen hypermethylated genes shared between the anal and cervical progression signatures, indicating that HPV-driven oncogenesis converges on a largely nonrandom and anatomically conserved pattern of epigenomic disturbance [11]. Critically, these methylation signatures classify high-grade intraepithelial lesions into cancer-like and normal-like categories that do not correspond neatly to conventional histological grading, indicating molecular heterogeneity within premalignant disease [11]. These findings support an epigenetic continuum of HPV-associated progression but should not, in isolation, be interpreted as direct demonstration of a field effect in histologically normal tissue.

### 5.3. Transcriptomic Reprogramming: Bulk and Single-Cell Evidence

Bulk transcriptomic profiling across the normal to intraepithelial neoplasia to carcinoma continuum has identified genes whose expression changes in a stepwise fashion that mirrors histological progression. Weighted gene coexpression network analysis of cervical samples spanning normal epithelium, cervical intraepithelial neoplasia, and invasive cancer identified FNDC3B and BPGM as genes whose expression is progressively dysregulated across every stage of this continuum, with upregulation of FNDC3B and downregulation of BPGM correlating with shorter overall and disease-free survival, indicating that these transcriptomic alterations are associated with HPV-related disease progression and clinical outcome [12]. Because this analysis compares stages along the normal to neoplasia continuum rather than spatially mapping histologically normal tissue surrounding a lesion, it is considered supportive progression evidence rather than direct evidence of field cancerization.

Single-cell and spatial transcriptomic technologies have substantially refined this picture by resolving cellular heterogeneity that bulk sequencing cannot capture. Integration of single-cell RNA sequencing with spatial transcriptomic mapping across normal cervix, HPV-infected normal cervix, high-grade squamous intraepithelial lesions, and invasive cancer has revealed that HPV infection of histologically normal cervical epithelium is already associated with distinct epithelial cell clusters and a graded remodeling of the local immune microenvironment, progressing from an initially reactive immune state toward progressive dysregulation and eventual immune exhaustion as the tissue advances toward malignancy [13]. This spatiotemporal atlas provides human tissue evidence consistent with early field-like remodeling, because molecular and cellular differences were detectable in histologically normal HPV-infected cervical epithelium in the absence of conventional morphological evidence of dysplasia. At still finer resolution, single-cell transcriptomic analysis of HPV16-infected epithelial organoids has identified a previously undescribed keratinocyte subpopulation, termed HPV-induced differentiation-dissonant epithelial nonconventional (HIDDEN) cells, that forms an aberrant superficial compartment dependent on overexpression of the transcription factor ELF3 and that is not present in uninfected epithelium [14]. This reprogrammed subpopulation persists across multiple stages of human carcinogenesis, being detectable in cervical intraepithelial neoplasia and in HPV-positive head and neck squamous cell carcinoma, and its associated biomarker signature correlates with worse clinical outcome when examined in large public tumor datasets, providing mechanistic evidence that HPV can establish an altered cancer-associated epithelial cell state and supporting the potential relevance of this cellular reprogramming to multistep HPV-associated carcinogenesis [14].

### 5.4. Metabolic Reprogramming as Mechanistic Support

Metabolic reprogramming toward aerobic glycolysis is a well-established hallmark of established cancers, and emerging evidence indicates that HPV-positive cells acquire a distinct metabolic phenotype even in the absence of overt malignant transformation. Comparative metabolomic profiling of HPV-positive and HPV-negative cervical cell lines using high-resolution mass spectrometry identified several hundred differentially abundant metabolites, with HPV-positive lines displaying features consistent with a Warburg-like metabolic shift together with heightened purine salvage pathway activity, changes attributed in part to the direct action of the E6 oncoprotein on host metabolic regulators [15]. These findings demonstrate HPV-associated metabolic reprogramming under experimental conditions and therefore provide mechanistic plausibility for metabolic alterations during field formation. However, because the evidence derives from cervical cancer cell lines rather than spatially sampled histologically normal human epithelium, it should be regarded as supportive rather than direct evidence of field cancerization [15].

### 5.5. Immune Microenvironment Remodeling

**Figure 1:**
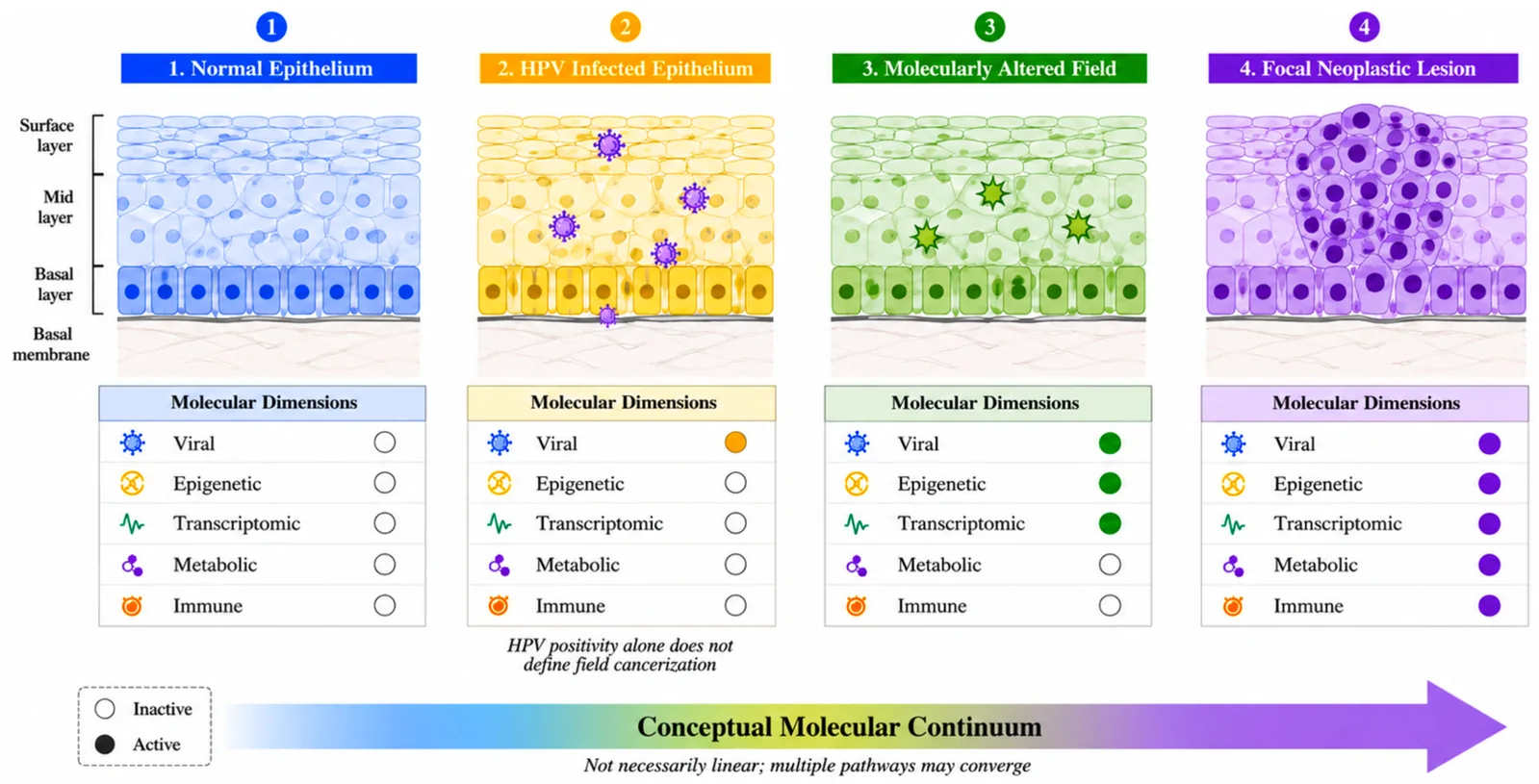
Conceptual framework distinguishing HPV infection from HPV-induced field cancerization. HPV infection of otherwise histologically normal epithelium is shown as an early biological state but is not considered sufficient to define a cancerized field. In this review, field cancerization refers to spatially extended molecular alterations within histologically normal epithelium, whereas premalignant or neoplastic lesions represent subsequent stages of progression. Epigenetic, transcriptomic, metabolic, and immune alterations shown in the figure represent molecular dimensions with varying levels of direct human tissue and mechanistic evidence. The schematic represents a conceptual model and does not imply that each molecular alteration has been longitudinally demonstrated to precede or causally initiate neoplastic transformation. Abbreviation: HPV, human papillomavirus. Created in BioRender. Kurt, B. (2026) https://BioRender.com/upajkg5.

Persistence of HPV infection depends critically on the capacity of infected keratinocytes to evade innate and adaptive immune surveillance, and similar immune evasion mechanisms have been described across HPV-infected epithelium and HPV-associated lesions [4,16]. HPV oncoproteins downregulate chemokines and adhesion molecules required for Langerhans cell and dendritic cell recruitment, impair natural killer cell activation through suppression of type I interferon signaling, and promote a cytokine milieu, including interleukin ten, transforming growth factor beta, and prostaglandin E2, that favors regulatory T cell expansion and a shift from a type 1 to a type 2 helper T cell response [16]. This coordinated program of local immunosuppression, established during the productive phase of infection in histologically normal tissue, is thought to create the permissive microenvironment within which subsequent genetic and epigenetic alterations can accumulate without being eliminated by immune surveillance, thereby facilitating the clonal expansion required for field formation and, ultimately, malignant progression [16] (Figure 1). Collectively, these mechanisms provide a biologically plausible route through which persistent HPV infection may establish a permissive local environment for subsequent clonal expansion and field formation. However, much of this evidence is mechanistic and should not be interpreted as direct spatial demonstration of a cancerized field in histologically normal human tissue [16] (Figure 1). The evidence discussed across these molecular dimensions is summarized according to biological context and evidence type in Table 1.

## 6. Field Cancerization Across HPV-Associated Malignancies

The strength of evidence supporting HPV-associated field cancerization varies substantially among anatomical sites. In this review, the cervical evidence is considered the most substantial, oropharyngeal evidence supportive, anal evidence suggestive, and bladder evidence controversial and currently insufficient to establish a consistent HPV-driven field effect. These descriptors reflect the quantity, biological context, and directness of the available molecular evidence rather than formal evidence grading.

### 6.1. Uterine Cervix

The cervix remains the anatomical site at which HPV-induced field cancerization has been most thoroughly characterized, in part because of the accessibility of cervical tissue to longitudinal sampling and in part because the stepwise progression from normal epithelium through cervical intraepithelial neoplasia to invasive carcinoma provides a well-defined model system [11,12,13,14,15]. The cervix currently provides the strongest and most methodologically diverse body of evidence relevant to HPV-induced field cancerization, although the underlying studies represent different evidentiary contexts. Single-cell and spatial analyses incorporating histologically normal HPV-infected cervical tissue provide human tissue evidence consistent with early field-like remodeling [13]. Laser capture microdissection has also detected low levels of HPV DNA in histologically normal cervical epithelial cells distant from tumor margins, although these findings may reflect subclinical infection rather than a cancerized field [17]. In contrast, methylation and bulk transcriptomic studies predominantly characterize molecular progression across normal, premalignant, and invasive disease states [11,12], while organoid and cell-line studies provide mechanistic support for epithelial reprogramming and metabolic alteration [14,15]. Taken together, these data support a graded HPV-associated molecular continuum while preserving the distinction between HPV infection, premalignant progression, experimental mechanisms, and direct evidence of a spatially extended molecular field.

### 6.2. Oropharynx

In the oropharynx, field cancerization has historically been attributed to chronic tobacco and alcohol exposure across the upper-aerodigestive-tract mucosa, and this classical model correctly predicts the frequent occurrence of second primary tumors in smoking-related, HPV-negative disease [18]. The emergence of HPV-positive oropharyngeal cancer as a distinct clinical and molecular entity has raised the question of whether an analogous, virus-driven field also exists in this setting, providing human tissue evidence consistent with an HPV-associated molecular field in the oropharyngeal mucosa that is distinct from the classical carcinogen-associated pattern [9]. These observations further support the possibility that molecular abnormalities may extend beyond the histologically defined tumor boundary, although the available HPV-specific evidence remains more limited than that available for the cervix [8,9]. The emergence of HPV-positive oropharyngeal cancer as a distinct clinical and molecular entity has raised the question of whether an analogous virus-driven field exists in this setting. Peritumoral molecular mapping has identified patchy microRNA alterations in histologically normal mucosa surrounding HPV-positive tumors, providing human tissue evidence consistent with an HPV-associated molecular field [9]. Conversely, transcriptionally active HPV has not been consistently identified in tumor-free adjacent or distant oropharyngeal mucosa, arguing against the existence of a universal HPV-driven field. Accordingly, the available evidence remains supportive but more limited and heterogeneous than that available for the cervix.

### 6.3. Anal Canal

The anal canal shares with the cervix a squamocolumnar transformation zone that is particularly susceptible to HPV-driven neoplastic transformation, and the two sites demonstrate substantial molecular convergence. Longitudinal evidence has also emerged from a multicenter cohort of patients with previous high-grade anal lesions, in which higher ZNF582 and ASCL1 methylation levels were associated with subsequent progression to anal cancer [19]. These data support the biological plausibility of an analogous anal field effect, but because they primarily derive from comparisons across histological disease stages rather than spatial mapping of histologically normal anal epithelium, they do not independently establish a true anal cancerized field. Genome-wide methylation profiling has shown that anal and cervical carcinoma share seventeen hypermethylated genes within their respective progression signatures and that these signatures can segregate high-grade anal and cervical intraepithelial lesions into cancer-like and normal-like categories [11]. These findings demonstrate substantial molecular convergence between anal and cervical HPV-associated neoplastic progression and provide suggestive evidence for an analogous anal field effect. However, because the available evidence is derived primarily from a limited number of molecular progression studies rather than direct spatial mapping of histologically normal anal epithelium, a true anal cancerized field cannot yet be considered established.

### 6.4. Urinary Bladder: A Controversial and Exploratory Extension

The role of HPV in bladder carcinogenesis, and by extension the applicability of a field cancerization model to this site, remains considerably more contested than in the cervix, oropharynx, or anus. Meta-analytic estimates of HPV prevalence among bladder cancer specimens have varied widely across studies. An early pooled analysis of fifty-two studies reported an overall HPV prevalence of approximately seventeen percent among bladder cancer cases, most of high-risk genotypes, and concluded that HPV positivity was associated with an increased risk of bladder cancer [20]. A subsequent systematic review restricted to studies published over the following decade reported a broadly similar prevalence but, in contrast, found no statistically significant association between HPV infection and bladder cancer, a discrepancy attributed to differences in geographical region and HPV detection methodology across the included studies [21]. More recent case control evidence has tended to favor a positive association: a systematic review and meta-analysis of case control studies applying Cochrane and PRISMA methodology reported a strong association between HPV positivity and bladder cancer diagnosis [22], and a two-sample Mendelian randomization analysis incorporating genetic instruments for HPV E7 protein exposure similarly found an elevated pooled prevalence of HPV16 and HPV18 among bladder cancer cases, although it could not establish unequivocal causality [23]. Accordingly, current evidence for HPV-induced field cancerization in the urinary bladder should be regarded as controversial and insufficient to establish a consistent field effect. The available studies primarily address the association between HPV infection and bladder cancer rather than directly demonstrating spatially extended molecular abnormalities in histologically normal urothelium. Importantly, this uncertainty operates at two levels: the etiological contribution of HPV to bladder carcinogenesis itself remains unresolved, and evidence for a spatially extended HPV-induced molecular field in histologically normal urothelium is even more limited. The bladder should therefore be regarded as an exploratory extension of the HPV field cancerization hypothesis rather than an established HPV-driven anatomical model.

**Figure 2:**
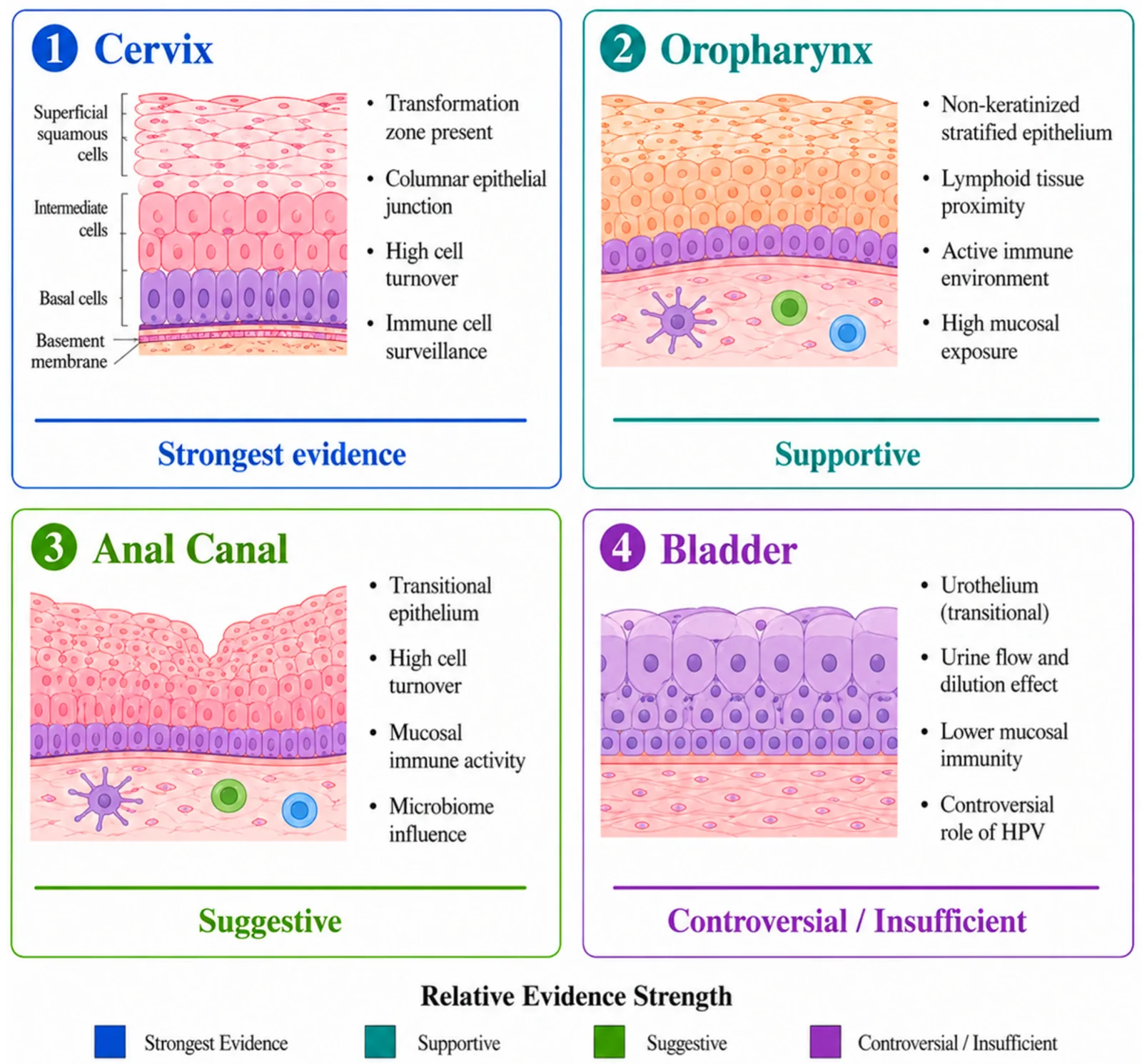
Comparative anatomical overview of evidence for HPV-associated field cancerization. Evidence strength is presented qualitatively according to the directness and breadth of the available molecular studies and does not represent a formal evidence-grading system. Cervical evidence is currently the most substantial, whereas oropharyngeal evidence is supportive, anal evidence remains suggestive, and bladder evidence is controversial and insufficient to establish a consistent HPV-driven field effect. For the urinary bladder, the designation “controversial/insufficient” reflects uncertainty both in the underlying association between HPV and bladder carcinogenesis and in the evidence for a corresponding field cancerization process. Abbreviation: HPV, human papillomavirus. Created in BioRender. Kurt, B. (2026) https://BioRender.com/ouuaf84.

The comparative distribution and site-specific molecular characteristics of field cancerization across these four organ systems are illustrated in Figure 2.

## 7. Methodological Approaches for Detecting the Field Effect

### 7.1. Tissue-Based Molecular Mapping

The most direct approach to characterizing field cancerization involves systematic molecular sampling of tissue surrounding a known lesion, an approach exemplified by the three-dimensional mucosal mapping studies described in Section 6.2, which combine radiological localization with biopsy-based genotyping and microRNA quantification to construct a spatially resolved model of the molecular field [9]. Although informative, tissue-based mapping is inherently invasive and therefore poorly suited to population-level screening, motivating the development of the minimally invasive approaches summarized below.

### 7.2. Single-Cell and Spatial Transcriptomics as Emerging Tools for Early Molecular Detection

Single-cell RNA sequencing (scRNA-seq), particularly when combined with spatial transcriptomic approaches, offers a high-resolution means of resolving epithelial and immune-cell states associated with HPV-related carcinogenesis. In cervical tissue, integrated single-cell and spatial analyses have identified distinct epithelial populations and changes in the local immune microenvironment across histologically normal HPV-infected tissue, high-grade lesions, and invasive cancer [13]. These observations suggest that single-cell approaches may help characterize early molecular changes and identify candidate biomarkers for further validation.

At present, however, scRNA-seq remains primarily a research and discovery tool rather than a clinically validated screening method. Its use in early detection is limited by the need for tissue sampling, technical complexity, cost, and the lack of prospectively validated diagnostic thresholds. Experimental HPV16-infected epithelial models further show that single-cell analysis can reveal previously unrecognized keratinocyte subpopulations associated with HPV-driven cellular reprogramming, supporting its value for mechanistic investigation and biomarker discovery [14].

### 7.3. DNA Methylation Biomarkers for Triage of HPV-Positive Individuals

DNA methylation assays have emerged as clinically relevant tools for risk stratification and triage among women who test positive for high-risk HPV. These assays primarily identify methylation patterns associated with HPV-driven neoplastic progression and the likelihood of high-grade disease; their clinical performance should not be interpreted as direct evidence that they specifically detect a spatially defined field cancerization process. Several methylation marker panels have now been validated in large, multicenter cohorts (Table 2). The FAM19A4 and miR124-2 (QIAsure; Self-screen B.V., Amsterdam, the Netherlands) methylation test, evaluated in a European multicenter study of 2384 HPV-positive women, achieved a sensitivity of seventy-seven percent and a specificity of seventy-eight-point-three percent for detection of cervical intraepithelial neoplasia grade three or worse, with a negative predictive value exceeding ninety-nine percent for invasive cancer among methylation-negative, HPV-positive women [24]. The ASCL1 and LHX8 methylation panel, evaluated in self-collected samples from the IMPROVE screening trial, achieved a sensitivity of seventy-three-point-three percent and a specificity of sixty-one-point-one percent for high-grade lesions, with performance in self-collected material closely approximating that obtained from clinician-collected samples, a finding with direct relevance for expanding screening access to underscreened populations [25]. The ZNF582 methylation marker has similarly demonstrated a strong correlation with lesion severity, with methylation levels rising progressively from normal tissue through increasing grades of cervical intraepithelial neoplasia to invasive cancer, supporting its candidacy as an additional triage biomarker [26]. Most recently, a real-world evaluation of the WID cervical intraepithelial neoplasia (qCIN) methylation test was conducted within a population-based Swedish screening cohort of 28,017 women, of whom 2377 were HPV positive; diagnostic performance estimates for the triage strategy were calculated within this HPV-positive subgroup [27]. Collectively, these methylation panels provide objective and reproducible approaches for identifying HPV-positive individuals at increased risk of high-grade cervical disease. Their clinical utility reflects association with HPV-driven molecular progression rather than specific validation as biomarkers of field cancerization. Demonstration of a true field effect would additionally require spatial evidence of corresponding molecular abnormalities in histologically normal epithelium.

### 7.4. Liquid Biopsy and Circulating HPV DNA

A parallel and promising investigational approach exploits the shedding of viral and host-derived cell-free DNA into the circulation, enabling detection of field-level and early neoplastic changes through a peripheral blood sample rather than direct tissue sampling. In cervical cancer, droplet digital polymerase chain reaction-based quantification of circulating cell-free HPV16 and HPV18 DNA has been shown to distinguish patients with active disease from healthy controls and to decline measurably following treatment, supporting its use as a prognostic and monitoring biomarker [28].

The clinical applications of circulating HPV DNA differ substantially according to cancer type and study setting and should therefore not be interpreted as directly comparable measures of early detection performance. In cervical cancer, Gupta et al. evaluated circulating cell-free HPV16 and HPV18 DNA using droplet digital PCR in patients with established disease and reported detection rates of 82.86% and 45.71%, respectively. Circulating HPV16 DNA levels correlated with tumor size and changed following treatment, supporting a potential role in disease burden assessment and monitoring rather than prediagnostic screening [28]. Conversely, circulating HPV DNA has shown limited sensitivity in pre invasive and very early invasive cervical disease, emphasizing that performance in established cancer should not be extrapolated to early detection [29]. Prospective cervical cancer data have similarly supported circulating HPV DNA as a marker of residual disease and relapse during post-treatment follow-up [30].

**Figure 3:**
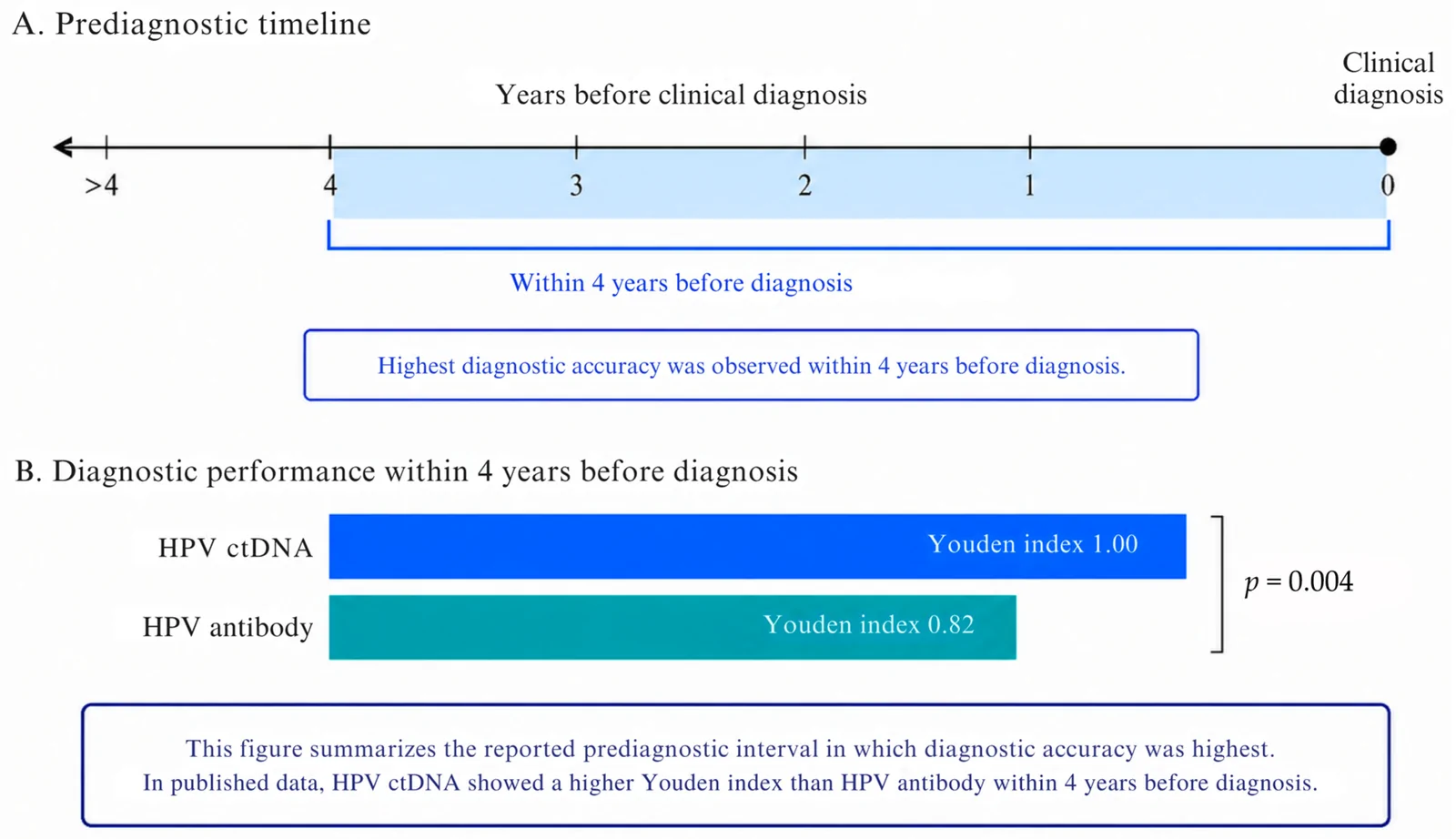
Prediagnostic detection of HPV-associated oropharyngeal squamous cell carcinoma using circulating tumor HPV DNA. In a case control cohort comprising 28 patients with HPV-associated oropharyngeal squamous cell carcinoma and 28 matched controls, a multifeature HPV whole-genome sequencing assay detected circulating tumor HPV DNA in 22 of 28 prediagnostic cases, corresponding to an overall sensitivity of 79% and a specificity of 100%, with a maximum lead time of 7.8 years before clinical diagnosis. Diagnostic accuracy was highest within four years before diagnosis. Application of a machine learning classifier increased overall sensitivity to 96% (27 of 28 cases) and extended the maximum reported lead time to 10.3 years. Values shown are directly reported by Das et al. and do not represent pooled or meta-analytic estimates. In the figure, blue represents HPV ctDNA and teal represents HPV antibody. Abbreviations: HPV, human papillomavirus; HPV ctDNA, circulating tumor human papillomavirus DNA. Created in BioRender. Kurt, B. (2026) https://BioRender.com/oxanmfc.

More direct evidence for prediagnostic detection has recently emerged in HPV-associated oropharyngeal squamous cell carcinoma. Das et al. analyzed plasma samples obtained 1.3 to 10.8 years before clinical diagnosis from 28 patients and 28 matched controls using a multifeature HPV whole-genome sequencing assay. Circulating tumor HPV DNA was detected in 22 of 28 prediagnostic cases, corresponding to an overall sensitivity of 79%, while all controls tested negative, yielding a specificity of 100%. The maximum lead time was 7.8 years, and diagnostic accuracy was highest within four years before diagnosis. Application of a machine learning classifier increased detection to 27 of 28 cases, corresponding to an overall sensitivity of 96%, and extended the maximum lead time to 10.3 years [31] (Figure 3). These findings should be interpreted as retrospective evidence of prediagnostic detectability rather than demonstration of a clinically effective screening strategy. Before population level or targeted screening can be considered, prospective validation is required to define appropriate target populations, positive predictive value in low-prevalence settings, the clinical consequences of false-positive findings, cost effectiveness, and whether earlier molecular detection ultimately improves patient outcomes. Clinical translation will also require standardization of preanalytical sample handling, analytical platforms, positivity thresholds, and reporting procedures, because variability across these factors may influence both false-positive and false-negative rates and complicate comparisons between studies.

## 8. Translational Relevance and Pathway to Clinical Implementation

Translation of molecular findings into clinical practice requires several distinct stages of evaluation rather than a direct transition from analytical detection to population screening. These stages include biological proof of concept, biomarker validation, prospective clinical validation, demonstration of clinical utility, and, ultimately, implementation within routine care. Progress at one stage does not necessarily imply that the requirements of the next have been met.

Among the approaches discussed in this review, cervical DNA methylation assays currently have the strongest clinical validation, particularly for triage of defined HPV-positive populations. However, their performance should not be interpreted as evidence of field cancerization-specific detection or as support for universal population screening. In contrast, prediagnostic detection of circulating tumor HPV DNA in oropharyngeal cancer remains promising but is based primarily on retrospective evidence and should therefore be regarded as an emerging biomarker approach rather than an established screening strategy.

The molecular evidence reviewed above carries direct implications for three interconnected areas of clinical practice [24,27]. First, with respect to screening, DNA methylation and circulating HPV DNA assays show potential for molecular risk stratification and early disease detection in selected clinical settings, but the current evidence does not support their routine use for population screening across HPV-associated cancers. Cervical methylation assays are further advanced for triage of HPV-positive individuals, whereas circulating HPV DNA approaches require prospective validation in clearly defined target populations before screening implementation can be considered [24,31].

Second, with respect to risk stratification, methylation and transcriptomic field signatures that segregate histologically similar lesions into cancer-like and normal-like categories suggest that current histological grading systems may inadequately capture the biological heterogeneity of intraepithelial neoplasia, and that molecular field profiling could allow clinicians to identify, among patients with a given histological grade, the subset at genuinely elevated risk of progression who warrant closer surveillance or earlier intervention [11]. Prophylactic HPV vaccination prevents persistent infection with vaccine-targeted high-risk HPV types and would therefore be expected to reduce downstream HPV-associated molecular alterations that may contribute to field formation. Population-based evidence further demonstrates that HPV vaccination is associated with a reduced risk of invasive cervical cancer [32]. Mathematical modeling of vaccination programs indicates that coverage of eighty percent among girls is projected to reduce the prevalence of persistent high-risk HPV infection by approximately sixty-five percent in vaccinated females, with additional herd protection extending to unvaccinated males, underscoring that primary prevention through vaccination and secondary prevention through molecular screening represent complementary rather than competing strategies for reducing the burden of HPV-associated cancer [33]. Accordingly, HPV-associated cancers are not considered examples of field cancerization solely because they are HPV positive; the field cancerization framework is applied only when molecular abnormalities extend beyond the focal lesion into histologically normal or tumor-adjacent epithelium in a spatially distributed manner. The purpose of using the field cancerization concept in this review is therefore not to reclassify all HPV-associated cancers, but to evaluate whether selected HPV-driven malignancies exhibit molecular changes that extend beyond the histologically defined lesion.

## 9. Critical Synthesis and Limitations

The evidence reviewed in this article converges on a model in which HPV-associated carcinogenesis is not adequately described as a purely focal process. The evidence reviewed in this article supports a spectrum of HPV-associated molecular alterations rather than a uniform body of direct evidence for field cancerization [11,16]. The strongest evidence for a true molecular field derives from studies demonstrating spatially distributed abnormalities in histologically normal HPV-positive or tumor-adjacent human epithelium, including peritumoral molecular mapping in the oropharynx and single-cell or spatial analyses of histologically normal HPV-infected cervical tissue [9,13]. By contrast, studies of premalignant lesions and established tumors primarily define the molecular continuum of HPV-associated neoplastic progression, while organoid, cell-line, and mechanistic studies provide biological explanations for how a field might arise. These categories are complementary but not interchangeable. Accordingly, HPV positivity or the presence of an HPV-associated molecular abnormality should not alone be interpreted as evidence that a cancerized field has been established.

This reconceptualization has several practical implications that extend beyond the individual findings summarized in the preceding sections [8,9]. Surgical and ablative treatment strategies that are planned solely with reference to the histologically visible margin of a lesion may leave behind molecularly altered tissue that retains elevated risk of recurrence or second primary tumor formation, a concern that is particularly relevant in the head and neck region, where field-level microRNA and epigenetic alterations have been shown to extend into tissue that appears grossly and microscopically unremarkable [8,9]. Similarly, screening programs that rely exclusively on morphological assessment, whether through cytology or colposcopic impression, are inherently limited in their capacity to detect the earliest molecular manifestations of field transformation, a limitation that the methylation-based and liquid biopsy approaches discussed above are specifically designed to overcome [24,25].

At the same time, several important limitations temper the strength of the conclusions that can currently be drawn. The evidence base is markedly uneven across the four anatomical sites considered in this review [11,15]. The cervical model is supported by the largest and most methodologically diverse body of work, encompassing bulk transcriptomic, single-cell, spatial, epigenetic, and metabolomic data obtained from large, well-characterized cohorts, whereas the oropharyngeal and anal literature, though directionally consistent with a field cancerization model, rests on a comparatively smaller number of studies with more limited sample sizes [20,23]. The bladder represents a case apart, where meta-analyses of HPV prevalence and its association with cancer risk have produced frankly discordant results, ranging from strong positive associations to an absence of statistical significance, and where methodological heterogeneity in HPV detection techniques across studies makes it premature to conclude that a genuine HPV-driven field cancerization process operates in the urothelium [20,21,22,23]. Readers should therefore interpret the bladder-related evidence summarized in this review with considerably greater caution than the evidence available for the other three sites.

Interpretation of field cancerization in the oropharynx is further complicated by potential confounding from tobacco and alcohol exposure, which may coexist with HPV positivity and independently generate carcinogen-associated molecular fields. Consequently, molecular abnormalities identified in peritumoral mucosa of HPV-positive tumors cannot automatically be attributed to viral effects alone unless relevant exposure histories and HPV-specific molecular features are adequately considered. Studies directly comparing HPV-positive and HPV-negative tumors, together with adjustment for smoking and alcohol exposure, are therefore particularly important for distinguishing virus-associated from carcinogen-associated field effects [9].

A further limitation concerns causality and temporality. Much of the molecular field evidence reviewed here derives from cross-sectional comparisons of tissue at different stages along the presumed normal-to-precancer-to-cancer continuum, an approach that can establish association but cannot by itself demonstrate that the molecular alterations observed in histologically normal tissue are causally necessary for, rather than simply correlated with, subsequent malignant transformation. Prospective, longitudinal studies that track individual patients or defined tissue fields over time, ideally incorporating serial molecular sampling alongside clinical outcome data, will be required to establish which field-level alterations are genuinely predictive of progression and which represent biologically inert bystander changes. The prediagnostic liquid biopsy data described earlier, in which archived plasma samples collected years before diagnosis were retrospectively analyzed, represent an important step in this direction, but comparable prospective validation across the other detection modalities discussed in this review remains an important priority for future research [28,31].

Finally, translation of the molecular findings reviewed here into routine clinical practice will require attention to feasibility, cost, and equity of access, particularly for liquid biopsy and next-generation sequencing-based assays that currently require specialized laboratory infrastructure not universally available, including in the low- and middle-income settings that continue to bear a disproportionate share of the global HPV-associated cancer burden [24,27]. More broadly, prospective validation in predefined target populations, assessment of positive predictive value in low-prevalence settings, characterization of false-positive consequences, cost-effectiveness analysis, and evidence that earlier detection leads to improved clinical outcomes remain necessary before these approaches can be considered clinically useful or ready for implementation.

## 10. Future Research Priorities

Future studies should prioritize longitudinal and spatially resolved sampling of histologically normal-appearing HPV-positive and tumor-adjacent tissues to determine whether candidate molecular alterations persist over time and are associated with subsequent neoplastic progression. Standardized operational definitions of field cancerization and reproducible criteria for defining molecular field boundaries will also be required to improve comparability across anatomical sites and study platforms. Integration of spatial transcriptomics, single-cell profiling, epigenomic analysis, and other spatial omics approaches may help resolve the cellular and molecular architecture of these fields at higher resolution. Candidate field biomarkers should additionally undergo prospective validation in clearly defined populations, with particular attention to whether they provide incremental predictive value beyond established factors such as HPV genotype, lesion grade, anatomical site, and conventional clinical parameters. Ultimately, future research should determine not only whether molecular fields can be detected, but whether their measurement improves risk stratification, guides surveillance or intervention, and produces clinically meaningful benefit.

## 11. Conclusions

Contemporary molecular evidence supports the biological plausibility of HPV-induced field cancerization and provides direct evidence of spatially extended molecular abnormalities in selected histologically normal HPV-positive or tumor-adjacent human tissues, particularly in the cervix and oropharynx. However, the evidence base is heterogeneous: studies of premalignant lesions and tumors primarily inform the continuum of HPV-associated progression, whereas organoid, cell-line, and mechanistic studies explain potential processes of field formation without independently demonstrating a cancerized field in vivo. The anal evidence remains suggestive but less directly established, while the proposed relationship between HPV and field cancerization in the urinary bladder remains substantially more uncertain. Realizing the full preventive potential of this evidence will require prospective validation of candidate field biomarkers, careful attention to the substantial site-specific heterogeneity in the strength of the existing evidence base, and continued investment in prophylactic vaccination to prevent persistent infection with vaccine-targeted high-risk HPV types and thereby reduce downstream HPV-associated carcinogenic changes. Taken together, the shift from a focal to a field-based understanding of HPV-associated carcinogenesis represents a meaningful step toward earlier detection and more effective prevention of one of the most significant infection-associated cancer burdens worldwide. Overall, current evidence is strongest for the cervix, supportive but more limited for the oropharynx, suggestive for the anal canal, and controversial for the urinary bladder.

## Figures and Tables

**Table 1:** Evidence relevant to HPV-induced field cancerization according to biological context and evidence type.

Anatomic Site	Evidence Context	Molecular Dimension	Key Finding
Oropharynx (direct)	Histologically normal peritumoral human tissue	Viral/microRNA	Patchy alteration of miR-21-5p and miR-155 detected in peritumoral mucosa surrounding HPV-positive tumors, distinct from the field surrounding HPV-negative tumors [9]
Head and neck (general) (supportive)	Tumor-adjacent/peritumoral human tissue; evidence not uniformly HPV specific	Multi-marker peritumoral	Recurrent dysregulation of CDKN2A/p16, MDM2, E2F2, ETS-1, MGMT and multiple microRNAs shared between tumor and adjacent peritumoral mucosa [8]
Cervix and anal canal (supportive)	Normal, premalignant, and carcinoma human tissue progression series	Epigenetic (DNA methylation)	Seventeen hypermethylated genes shared between cervical and anal progression signatures; segregates lesions into cancer-like and normal-like categories [11]
Cervix (supportive)	Normal, premalignant, and carcinoma human tissue progression series	Transcriptomic (bulk)	FNDC3B upregulation and BPGM downregulation across normal, intraepithelial neoplasia and cancer stages, correlated with survival [12]
Cervix (direct)	Histologically normal HPV-infected tissue, HSIL, and carcinoma; single-cell/spatial human tissue	Single-cell/spatial transcriptomic	Graded immune microenvironment remodeling from HPV-infected normal cervix through high-grade lesions to cancer [13]
Cervix and head/neck (organoid model) (experimental)	Organoid model with validation across human lesions and tumors	Cellular reprogramming	HIDDEN keratinocyte subpopulation dependent on ELF3 overexpression, detectable from intraepithelial neoplasia through HPV-positive carcinoma [14]
Cervix (cell-line model) (experimental)	Cell-line model	Metabolic	Warburg-like glycolytic shift and heightened purine salvage pathway activity in HPV-positive lines [15]
Multiple sites (cervix, head/neck) (experimental)	Mechanistic and experimental evidence	Immune microenvironment	Suppressed Langerhans cell and natural killer cell activity, regulatory T cell expansion, and a type 1 to type 2 helper T cell skew driven by HPV oncoproteins [16]

Abbreviations: HPV, human papillomavirus; DNA, deoxyribonucleic acid; HSIL, high-grade squamous intraepithelial lesion; HIDDEN, HPV-induced differentiation-dissonant epithelial nonconventional. Gene and protein symbols are presented according to standard nomenclature.

**Table 2:** DNA methylation biomarkers for triage of HPV-positive women in cervical cancer screening.

Marker/Test	Study Population	Sensitivity (CIN3/CIN3+, as Reported)	Specificity	Ref.
FAM19A4/miR124 2 (QIAsure)	European multicenter, *n* = 2384 HPV-positive women	77.0% (95% CI 71.3–82.2) for CIN3	78.3% (95% CI 76.4–80.0)	[24]
ASCL1/LHX8	IMPROVE self-sampling trial, *n* = 593 HPV-positive women	73.3% (95% CI 63.9–82.6) for CIN3+	61.1% (95% CI 56.9–65.4)	[25]
ZNF582	Case control study, *n* = 199 HPV-positive women	71.2% (95% CI 56.9–82.9), CIN3+	88.4% (95% CI 82.1–93.1)	[26]
WID qCIN + HPV16/18	Population-based Swedish screening cohort, *n* = 28,017 women; diagnostic performance evaluated among 2377 HPV-positive women	93.4% (95% CI 85.7–97.3), CIN3; 100% invasive cancer	60.7% (95% CI 58.5–62.9)	[27]

Abbreviations: HPV, human papillomavirus; HPV16/18, human papillomavirus genotypes 16 and 18; CIN, cervical intraepithelial neoplasia; CIN3, cervical intraepithelial neoplasia grade 3; CIN3+, cervical intraepithelial neoplasia grade 3 or worse; CI, confidence interval.

## Data Availability

No new data were created or analyzed in this study. Data sharing is not applicable to this article.
